# Hepatitis B virus infection among children aged 1– 5 years of Mayurbhanj district (Odisha), India: A prospective study protocol

**DOI:** 10.1371/journal.pone.0317621

**Published:** 2025-05-29

**Authors:** K. Divyasree Bhat, Haimanti Bhattacharya, Yuvaraj Jayaraman, Srikanta Kanungo, Matrujyoti Pattnaik, Sanghamitra Pati, Debdutta Bhattacharya

**Affiliations:** 1 Department of Microbiology & One Health, ICMR- Regional Medical Research Centre, Bhubaneswar, Odisha, India; 2 The Tamil Nadu Dr MGR Medical University, Chennai, Tamil Nadu, India; 3 ICMR-National Institute of Epidemiology, Chennai, Tamil Nadu, India; Siksha O Anusandhan University School of Pharmaceutical Sciences, INDIA

## Abstract

**Background:**

Over past few years, global healthcare landscape has seen an increased recognition of the significance of infectious diseases, especially those affecting vulnerable populations such as young children, one of these diseases, Hepatitis B has emerged as a notable public health concern. Hepatitis B is highly transmissible during early years of life and may lead to complications like long-term chronicity, cirrhosis of liver and the progression of hepatocellular carcinoma. To address this growing concern, there is a pressing need for community-based studies to assess the prevalence, transmission patterns, risk factors, and potential interventions for Hepatitis B among children below the age of five. These studies should also assess the community’s knowledge and practices regarding Hepatitis B and immunization.

**Methods:**

The cross- sectional study will include a sample size of 2700 children aged between 1 to 5 years of Mayurbhanj district, Odisha. The serum samples will be tested for Hepatitis B surface antigen (HBsAg) and Hepatitis B surface antibody (anti-HBs). A detailed questionnaire will be prepared after focus group discussions with caregivers and healthcare workers to describe their knowledge and practices regarding HBV disease, associated risk factor and immunization.

**Discussion:**

The prospective sero-epidemiological study conducted within the community will assist in determining the prevalence of Hepatitis B within the vulnerable population. It will provide valuable insights for monitoring the current status of Hepatitis B in Mayurbhanj district and contribute to enhancing ongoing efforts in vaccination, control, and eradication of Hepatitis B.

## Introduction

Untreated Hepatitis B is one of the leading causes of liver cirrhosis and hepatocellular carcinoma accounting for up to 80% of cases worldwide [1]. The majority of the disease impact of HBV infection stems from acquiring infections before the age of five [1]. Globally, there were 1.3 million reported deaths attributed to viral hepatitis, with approximatly 254 million individuals estimated to be living with chronic Hepatitis B infection in 2022 [1]. In 2022, the World Health Organization estimated that Hepatitis B resulted in around 1.1 million deaths, predominantly attributed to liver cirrhosis and hepatocellular carcinoma [1]. The global burden included 2.9 million people living with chronic hepatitis B infection, with an annual reporting of 1.5 million new infections [1]. The World Health Organization’s assessment indicates that 1.3% of children under 5 years old are affected by HBV infection [2].

Hepatitis B is primarily transmitted through vertical transmission during childbirth, especially in high-prevalence areas, and horizontal transmission in early childhood through close contact with infected individuals [3]. Other significant causes include unprotected sexual contact, unsafe injections, contaminated blood transfusions, and organ transplants. Risk factors also include non-sterile tattooing, body piercing, and traditional practices like scarification [4]. Healthcare workers face exposure through needle-stick injuries, while household transmission occurs via shared personal items [5]. Although breastfeeding is not a direct cause, perinatal transmission during delivery is a major concern [3].

India holds an intermediate position in the global endemic landscape of Hepatitis B virus (HBV) infection with a prevalence of Hepatitis B surface antigen (HBsAg) ranging from 3% to 4.2% with 40 million HBV carriers [6]. HBV has been implicated in the cause of up to 80% of cases of Hepatocellular Carcinoma (HCC) [7]. The virus optimizes its life cycle to enable prolonged persistence in liver tissue by forming a plasmid-like covalently closed circular DNA structure [8]. Persistent chronic HBV infection in liver tissue is linked to increased oxidative damage in hepatocytes, inflammation of the liver driven by the immune system, and the development of cancer [9,10].

The course of HBV infection is influenced by the host’s age at the time of infection [11]. The likelihood of developing acute hepatitis and its transition to chronic infection exhibits an inverse correlation with the host’s age [12]. Infections contracted during infancy are often asymptomatic, with over a 90% probability of progressing to chronic infection [13]. About 20% of children aged 5 years develop chronic infection [14]. However, beyond the age of 5 years and especially in adults, over 90% develop acute hepatitis and successfully eliminate the virus within a six-month period [14].

According to the National Viral Hepatitis Control Program Operational Guidelines from the Ministry of Health and Social Welfare the prevalence of Hepatitis B among the general population in India ranges from 1.1% to 12.2% [15]. High risk for infections of Hepatitis B includes children from vertical transmission i.e maternal transmission, recipients of multiple blood or blood products transfusion, especially children or adults who undergo treatments for infectious diseases such as HIV, Kala azar, hemodialysis patients, drug users, tattoos, female sex workers and infected people’s sexual partners [16]. Despite the prevalence of negative disease outcomes, a significant number of patients are diagnosed at an advanced stage, often with hepatocellular carcinoma, with Hepatitis B being the primary contributing factor [17]. In India, Hepatitis B vaccination was initiated in 2002, initially focusing on urban areas encompassing 14 metropolitan cities. Subsequently, in 2003, the program expanded to include 33 more rural districts [18]. The Universal Immunization Program (UIP) recommended providing children with a series of three doses of the Hepatitis B vaccine, in addition to immunizations for six other diseases (Polio, Diphtheria, Pertussis, Tetanus, Tuberculosis, and Measles) that can be prevented through vaccination [19]. The present immunization schedule in India incorporates a birth dose administered within a 24-hour timeframe for every institutional delivery to thwart perinatal transmission. In addition to the birth dose, newborns receive three booster doses at 6, 10, and 14 weeks, along with oral poliovirus (OPV) and Diphtheria, Pertussis, Tetanus (DPT) vaccines, ensuring comprehensive immunization against Hepatitis B and addressing the needs of non-institutional births [19].

Booster doses play a critical role in maintaining long-term immunity, especially in individuals who may not have achieved optimal protection after the initial vaccination series [20]. The booster dose ensures that immunity remains high, providing continued protection against Hepatitis B infection [21]. Studies have shown that the booster dose stimulates a robust immune response, even in those with reduced antibody levels, reinforcing the protection and reducing the risk of breakthrough infections [22,23].

Hepatitis B vaccination coverage in India varies widely. According to a 2019 study conducted across 640 districts in India, 110 districts reported immunization coverage rates below 40% [24]. In the northern and northwestern regions of India, child immunization rates are less than 20% in 11 districts [25]. Previous research indicated that approximately 17 districts in India have achieved coverage rates exceeding 90% for complete child immunization with the three doses of the Hepatitis B vaccine [26]. Conversely, in a few districts, the coverage is as minimal as 5%. Among the 640 districts, only two districts in Punjab and one district in Kerala demonstrated a full 100% coverage rate for Hepatitis B vaccination [24,27]. However, incomplete data on coverage of Hepatitis B vaccination in India limits the estimation of the exact disease burden.

The current study protocol aims to estimate the Hepatitis B virus disease burden in children aged 1 to 5 years in India. Additionally, it seeks to compare the current prevalence of Hepatitis B virus infection with the immunization status and coverage in the district predominantly inhabited by tribal communities of Mayurbhanj district in the state of Odisha. The study seeks to address significant gaps in data regarding HBV prevalence and vaccination coverage in underserved populations especially in tribal regions of Mayurbhanj. Previous research has examined overall vaccination coverage and disease burden across India, however there remains a lack of district-specific studies, particularly in tribal regions where immunization access and healthcare infrastructure are often limited.

## Methodology

### Study design

**Fig 1:**
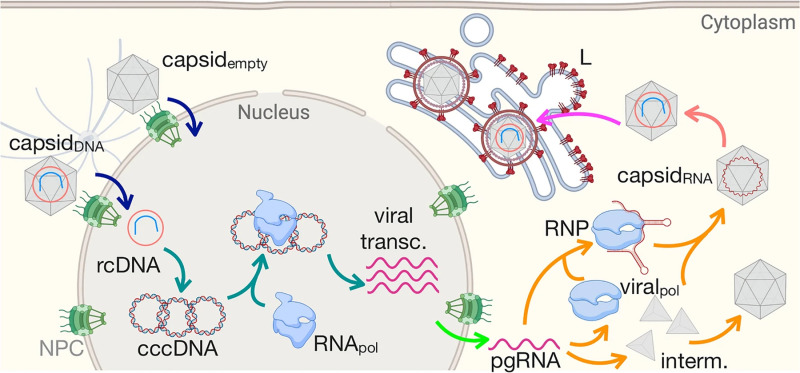
Hepatitis B virus life cycle [28].

The cross-sectional population under consideration comprises all residents within the specified age group (1 – 5 years) in the study district. The key outcome to be measured is the prevalence of Hepatitis B, determined by evaluating the positivity of HBsAg among the study participants. HBsAg positivity acts as an indicator of the disease within the selected population. Hepatitis B virus life cycle is depicted in Fig 1 [28].

### Study setting

**Fig 2:**
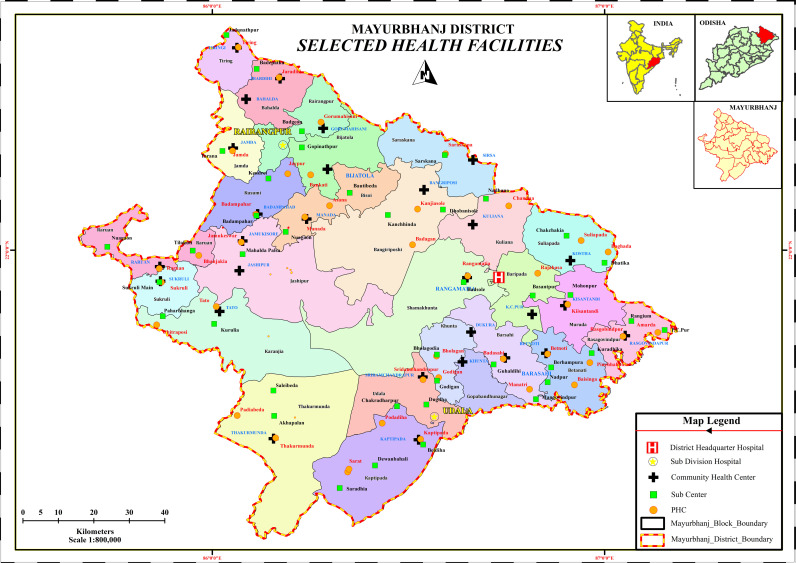
Map of Mayurbhanj district with selected Health Centers for the study.

This cross-sectional study aims to assess the prevalence and immunization status of Hepatitis B among children aged one to five in Mayurbhanj district, Odisha. Mayurbhanj, a landlocked district spanning 10,418 square kilometers, is located on the northern boundary of the state, with its district headquarters in Baripada. It shares borders with the Medinipur district of West Bengal to the north, Jharkhand’s Singhbhum district, to the west, Baleshwar and Keonjhar district to the south (Fig 2). More than 39% of the entire land area is covered by forests and hills. As of the 2011 census, the district’s population is approximately 2.5 million comprising 1.25 million males and 1.26 million females. The total number of children aged one to five years is 202,212 and the district hosts approximately 102 primary healthcare centers [29].

### Sample size

The target population for this study will consist of children aged between 1 to 5 years who are permanent residents of Mayurbhanj district. The sample will be selected through a multi-stage sampling technique. Each subcentre in the district will be treated as a cluster, and in the first stage the selection of subcentres will be based on a population proportional to the desired sample size. In the second stage, study participants within each selected subcentre will be chosen using simple random sampling. The sample size for seroprevalence was calculated to be 2700, considering the estimated population size as 202,212, 2.1% seropositivity, absolute precision + /-0.7%, with confidence level of 95% and 10% non- response rate [30]. The sample size was calculated using Open Epi, Version 3, an open-source calculator available on the internet.

### Sampling method and selection of participants

**Fig 3:**
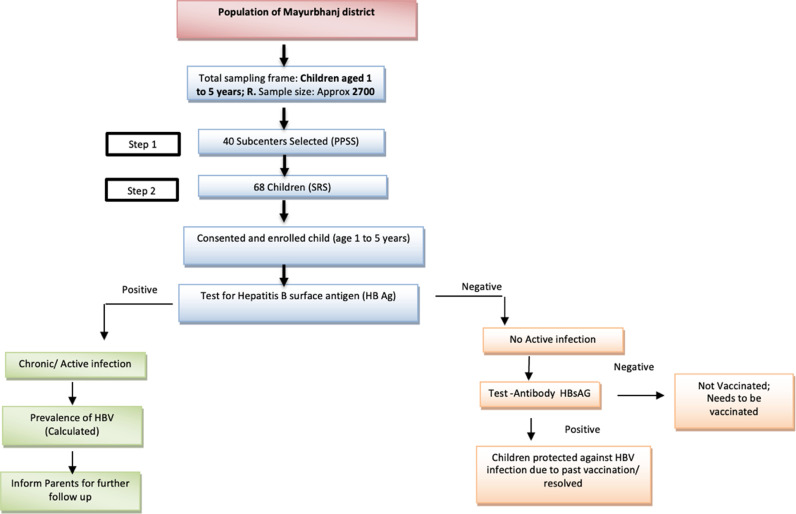
Sampling method for the study.

The research will utilize a 2- stage cluster sampling approach. In the initial stage, 40 clusters will be chosen with a population proportional to the sample size. Subsequently, in the second stage, 68 study participants will be randomly chosen from each cluster through a simple random sampling method (Fig 3).

### Study timeline

The participants recruitment for the quantitative study will begin from 1^st^ November 2024 and will continue until 30th April 2025. The initial version of this study protocol was submitted prior to the commencement of data collection. The data collection phase will continue until the target sample size is reached. Data analysis will begin once all data has been gathered.

### Sample collection procedure

**Fig 4:**
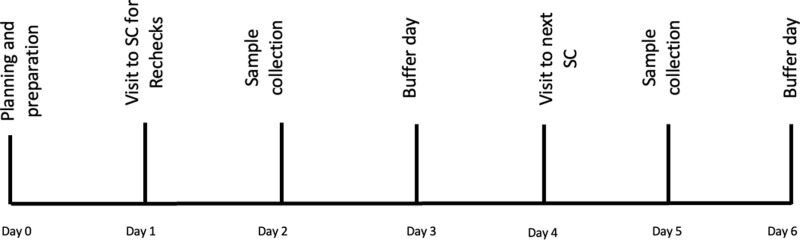
Sample collection schedule (week- wise).

After obtaining parental consent, the Accredited Social Health Activist (ASHA) or Auxiliary Nurse Midwife (ANM) will notify parents about the sample collection date at the nearby Primary Health Centre (PHC) or Village Health and Nutrition Day (VHND) (Fig 4). The blood sample will be collected using standard protocol for blood collection [31].

### Sample processing

Blood samples will be collected using gel vacutainers to perform serological tests. Within 12 hours of collection, the samples will be transported to the nearest health facility, where the serum will be separated through centrifugation at 3000 revolutions per minute (RPM) for a duration of 10 minutes. The serum aliquots will be stored at -20°C at the Department of Microbiology, District Hospital, Baripada. Subsequently, the serum samples will be transported to the laboratory, while maintaining proper cold chain for further testing and analysis.

### Laboratory investigations

1. HBsAg: HBsAg or Hepatitis B surface antigen, serves as the initial marker for acute infection and can also indicate chronic infection if its presence lasts longer than over six months. This proves valuable in both the diagnosis of HBV infection and the screening of blood. Samples testing negative for HBsAg will undergo additional testing for Anti-HBs IgG.
2. Anti- HBs IgG: The specific antibody targeting the Hepatitis B surface antigen. Its emergence within 1 - 4 months following the symptom’s onset signifies clinical recovery and further strengthening the immunity to HBV. Anti-HBs has the capability to neutralize HBV, offering protection against HBV infection.

### Screening for HBV prevalence

Screening of HBV infection will be done using serological testing for Hepatitis B surface Antigen (HBsAg) (J Mitra Pvt Ltd; sensitivity 100%, specificity 100%) and Hepatitis B surface antibody (Anti HBs) (DIAPRO, Italy; sensitivity 100%, specificity 100%) by performing Enzyme Linked Immunosorbent Assay (ELISA) following manufacturer’s instruction. All the serological testing will be carried out at the laboratory of ICMR- Regional Medical Research Centre, Bhubaneshwar.

### Data collection

#### Demographic and vaccination history.

**Fig 5:**
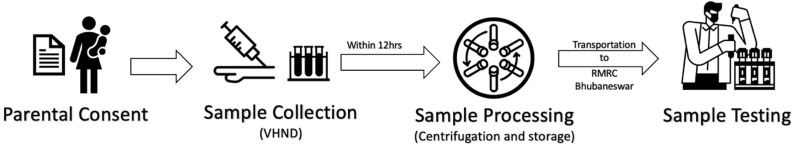
Overview of data collection and testing.

Demographic data of the children will be collected at the time of collection of blood sample (Fig 5). The child’s vaccination history will be obtained from the vaccination cards provided by the parents, and it will be crosschecked with the vaccination registers maintained by the respective ASHA/ANMs of the subcentres.

#### Focus group discussion and questionnaire development.

Focus Group Discussion (FGD) will be conducted with parents of 1 to 5year-olds residing in the villages/ towns of Mayurbhanj district to collect information on knowledge and attitudes regarding Hepatitis B disease and immunization. 20 focus group discussions with each FGD consisting of at least 6 to 8 parents/ guardians and five focus group discussions will be conducted among ASHA and ANM workers of the selected subcentres. All the information collected will be used to construct a quantitative questionnaire for administration to the parents/guardians of the children participating in the study. One in-depth Interview (IDI) will be conducted with the District Immunization Officer based on the summary of findings of FGD with community, field staff and Medical Officer and also based on the findings of the situation analysis. This will be based on the inputs gained from the FGD with ASHAs and Medical Officers on programmatic issues relating to Hepatitis B vaccination in the district. The developed questionnaire will be translated into the local language, back translated to check for errors, validated and pilot tested which will be further administered to the parents during sample collection.

#### Situation analysis of hepatitis B immunization in the district.

Proforma will be developed for data extraction from the registers available at the district headquarters. Past 3 years data will be collected which includes number of children catered by the ASHA, number of vaccines indented, number of children who received complete or incomplete immunization as per schedule. The District Immunization Officer of Mayurbhanj will be consulted on information about vaccine supply and stocks, stock maintenance at PHCs and district level and wastages if any.

### Data analysis plan

Descriptive statistics like mean, standard deviations and proportions will be calculated for the quantitative data. The weighted prevalence of Hepatitis-B infection among 1 to 5 years children will be estimated in percentage with 95% confidence intervals. Immunization coverage also will be calculated as weighted percentage with 95% CI. The proportion of children with protective level of HBV immunity response will also be calculated. Odds ratios for non- immunization will be calculated for the associated factors. Statistical analysis will be carried out in STATA v17.0. The district map was made using QGIS v3.34 which is freely available in the internet. Focus group discussions (FGDs) and In-depth interviews (IDIs) will be digitally recorded and translated into the English language. Data will be analyzed using content analysis. Key units relevant to the study objectives will be identified from the original transcripts. These units will then be condensed and coded. The codes will be grouped together, and the primary theme will be derived from these categories. Strengths, Weaknesses, Opportunities and Threats (SWOT) will be identified for HBV immunization coverage in Mayurbhanj district, Odisha.

### Ethics declarations

The study will adhere to the National Ethical Guidelines for Biomedical Research involving Human Participants issued by The Indian Council of Medical Research in 2017. All parents or guardians of the children will carefully read and sign the informed consent. The study received approval from the Institutional Ethics Committee at ICMR- Regional Medical Research Centre, Bhubaneshwar (EC Ref no: ICMR-RMRC.IHEC-2022/113) and State Ethics Board of Odisha (Ref to vide Letter no 18003/ MS- 2- IV- 04/2020 (PT-1), Bhubaneshwar dated 13-09-2022) of Directorate of Health Services, Govt of Odisha).

## Discussion

The state health authorities have highlighted that the high prevalence of persistent Hepatitis B in Odisha is a significant health concern. It results in significant rates of illness, death, and financial burden [30]. Hepatitis B presents a substantial worldwide public health concern, and the selected district is home to a diverse population, including many tribal communities. Tribal communities often reside in remote or rural areas, far from modern healthcare facilities [31–34]. Geographical isolation, inadequate transportation, and communication challenges hinder their access to medical services, which may significantly contribute to the disease burden among young children [31].

Hepatitis B in children often progresses silently, showing few or no symptoms until the disease has already advanced. As a result, parents and caregivers may not recognize the infection’s presence, leading to delayed diagnosis and missed opportunities for timely medical intervention. Early testing can help identify asymptomatic infections and prevent further transmission [35,36].

In India, it is essential to understand that vaccination efforts are ongoing and subject to changes and improvements based on evolving public health needs and priorities. Efforts are being made to address barriers such as limited healthcare access, low awareness, and vaccine hesitancy in certain communities. Between 2008 and 2016, vaccine coverage for Hepatitis B in India witnessed a rise from 28.9% to 62.8% [24]. It is also understood from past studies that infants and young children are particularly vulnerable to Hepatitis B infection, and they have a higher likelihood of developing chronic infections if exposed at an early age [29]. Early immunization helps build immunity before potential exposure, reducing the risk of chronic infection and its long-term consequences.

These studies are essential, as they provide insights into the vaccination coverage and compliance rates in the target population. Low vaccine coverage or incomplete vaccination schedules can increase children’s risk of infection. Some individuals may not develop sufficient immunity despite vaccination, especially in certain high-risk populations [34]. Studying the prevalence of Hepatitis B in vaccinated children can help assess the vaccine’s effectiveness and identify any potential gaps in protection. Studying the prevalence allows researchers to evaluate the immune response of vaccinated children. Some children may mount a weaker immune response, and understanding this response can help optimize vaccination strategies.

Studies have suggested that mother-to-child or father-to-child transmissions may be one of the reasons for vaccine failure. Another possible cause of vaccine failure may be attributed to non- response or hypo response to the vaccine [35]. Studying the prevalence of Hepatitis B will allow the researcher to assess the effectiveness of vaccination program. If a significant number of vaccinated children are found to have Hepatitis B, it raises the concerns about the vaccine’s efficacy or the need for a booster dose. Secondly, if any individual contracts the disease despite receiving the recommended dose, this study can help in planning further studies in analysing the specific strains of virus responsible for breakthroughs. Furthermore, understanding the prevalence of Hepatitis B among this age group helps public health authorities to identify high risk areas or communities. By pinpointing regions with a higher incidence or prevalence of disease, targeted vaccination campaigns and awareness programs can be implemented to reduce transmission rates. Additionally, healthcare resources can be allocated more efficiently to address the needs of these vulnerable populations.
